# Influence of Parent Concrete Properties on Compressive Strength and Chloride Diffusion Coefficient of Concrete with Strengthened Recycled Aggregates

**DOI:** 10.3390/ma13204631

**Published:** 2020-10-16

**Authors:** Jingwei Ying, Zewen Han, Luming Shen, Wengui Li

**Affiliations:** 1 College of Civil Engineering and Architecture, Guangxi University, Nanning 530004, China; 2 School of Civil Engineering, The University of Sydney, Sydney, NSW 2006, Australia; luming.shen@sydney.edu.au; 3 Key Laboratory of Engineering Disaster Prevention and Structural Safety of China Ministry of Education, Guangxi Key Laboratory of Disaster Prevention and Engineering Safety, School of Civil Engineering and Architecture, Guangxi University, Nanning 530004, China; ww_0321@163.com; 4 School of Civil and Environmental Engineering, University of Technology Sydney, Sydney, NSW 2007, Australia; Wengui.Li@uts.edu.au

**Keywords:** recycled coarse aggregate, pore structure, chloride diffusion, carbonation, nano-SiO_2_ slurry

## Abstract

Parent concrete coming from a wide range of sources can result in considerable differences in the properties of recycled coarse aggregate (RCA). In this study, the RCAs were obtained by crushing the parent concrete with water-to-cement ratios (*W/C*_parent_) of 0.4, 0.5 and 0.6, respectively, and were strengthened by carbonation and nano-silica slurry wrapping methods. It was found that when *W/C*_paren_ was 0.3, 0.4 and 0.5, respectively, compared with the mortar in the untreated RCA, the capillary porosity of the mortar in the carbonated RCA decreased by 19%, 16% and 30%, respectively; the compressive strength of concrete containing the carbonated RCA increased by 13%, 11% and 13%, respectively; the chloride diffusion coefficient of RAC (*D*_RAC_) containing the nano-SiO_2_ slurry-treated RCA decreased by 17%, 16% and 11%; and that of RAC containing the carbonated RCA decreased by 21%, 25% and 26%, respectively. Regardless of being strengthened or not, both *D*_RAC_ and porosity of old mortar in RCAs increased with increasing *W/C*_parent_. For different types of RCAs, *D*_RAC_ increased obviously with increasing water absorption of RCA. Finally, a theoretical model of *D*_RAC_ considering the water absorption of RCA was established and verified by experiments, which can be used to predict the *D*_RAC_ under the influence of different factors, especially the water absorption of RCA.

## 1. Introduction

In the process of urbanization, natural sand and stone resources for construction are rapidly decreasing, especially in China, and their prices have risen rapidly in recent years [1]. From an economic point of view, construction enterprises are forced to strengthen the recycling of waste concrete. For example, in large cities such as Beijing, Shanghai, and Shenzhen in China, many waste concrete treatment plants have been built, and recycled coarse aggregate (RCA) has been used to construct buildings and road pavement [2]. However, because the properties of RCA are generally worse than those of natural aggregate, for example, the crushing index and water absorption of RCA are higher [3], the use of RCA in concrete structures may present new problems with respect to mechanical performance and durability. Chloride diffusivity is one of the critical factors affecting concrete durability and is influenced by many parameters such as porosity [4], which is influenced by the water-to-cement ratio (*W/C*) [5]. The resistance to chloride penetration in RCA concrete (RAC) decreases as the RCA content and the *W/C* increase [6]. Compared to natural aggregates, RCAs have greater porosity and thus more significant water absorption, owing to the existence of old mortar [7]. Some researchers [8,9,10] have investigated the chloride permeability of RAC and summarized some essential characteristics. For example, Rajhans et al. [8] observed that self-compacting RAC prepared with a two-stage mixing approach could result in improved chloride diffusion resistance properties. Ma et al. [9] investigated the chloride permeability of recycled powder concrete, and suggested that the recycled powder replacement ratio in concrete should be below 30. In addition, Wu et al. [10] developed a multiscale digital image-driven stochastic method to analyze chloride ion diffusion in RAC. Therefore, the property of RCA has an important impact on the durability of RAC, which is very important for the design of RAC structure in coastal environment and deicing salt environment.

The properties of RCAs from various sources are different, which in turn affects the performance of RAC [11,12,13]. For example, Liu et al. [11] and Zhou et al. [12] found that concrete made with different types of RCAs has different shear strength, compressive strength, and flexural strength. Abdulla [13] investigated the properties of three main constituents within RCAs: unbound stone, crushed concrete, and crushed brick, and found that the performance of the concrete containing each type of RCA was largely influenced by the aggregate nature and quality, in addition to the attached mortar content. Some researchers have obtained different types of RCA through the carbonation method [14,15] or nano-slurry treatment [16,17,18] and have carried out the performance comparison between them. For example, Xuan et al. [14] carbonated a new type of RCAs derived from a designed concrete mixture, and an old type of RCA sourced from demolished old buildings. Their results showed that the compressive strength of the concrete with carbonated RCAs was improved by 6.6% and 22.6% compared to that with non-carbonated RCAs, respectively. Zhang et al. [15] carbonated RCA sourced from crushed stone and gravel in concrete, and the compressive strength of the concrete was improved by 6% and 10%, respectively. Furthermore, the nano-SiO_2_ slurry treatment method was used by Zhang et al. [16] to modify RCA sourced from RCA plants, and the result showed that the concrete compressive strength was improved by 35%. Singh et al. [17] investigated the modification effect of nano-SiO_2_ slurry on the interfacial transition zone (ITZ) of RCA resulting from Infrastructure Leasing and Financial Services (IL&FS), Burari, India, and suggested that nano-SiO_2_ slurry mediation accelerated hydration products and densified both the new and old ITZs, leading to substantial improvement in macro properties of RAC. As a result, the carbonation method and nano-SiO_2_ slurry treatment are considered potential solutions to the enhancement of RAC. Some scholars have carried out a series of studies on chloride ion diffusion of RCA concrete [3,8]. For example, Ma et al. [3] tested the chloride diffusion properties of recycled concrete under the coupling of the freeze–thaw cycle, high temperature and mechanical damage, and Rajhans et al. [8] prepared self-compacting concrete resistant to chloride ion erosion. To predict chloride diffusion, some scholars have proposed different models [19,20]. For example, Yu et al. [19] proposed a prediction model of chloride ion diffusion coefficient considering material properties, including water-to-cement ratio, fly ash and slag content, etc. Wang et al. [20] proposed a chloride diffusion prediction model considering the properties of coarse aggregate and reinforcement. From the above analysis, it can be concluded that the chlroide difusion properties of RCA is affected by the source and properties of parent concrete, and the strengthening method of RCA. Therefore, it is necessary to further investigated the influence of the property of parent concrete on the chloride diffusion properties of RAC.

In this study, according to the different effects of the water-to-cement ratio of parent concrete on the properties of RCA, comprehensive methods such as carbonation and nano-silica slurry were used to strengthen the RCA. The effects of strengthening methods and water-to-cement ratio on the properties of RCA were analyzed by comparing the pore structures, compressive strengths, and chloride diffusion coefficients of different types of RAC, and a composite model for predicting the chloride diffusion coefficient of RAC was proposed. It contributes to the understanding of the influence of RCAs from different sources and different RCA strengthening methods on the compressive strengths and chloride diffusion properties of RAC.

## 2. Experimental Program

**Figure 1:**
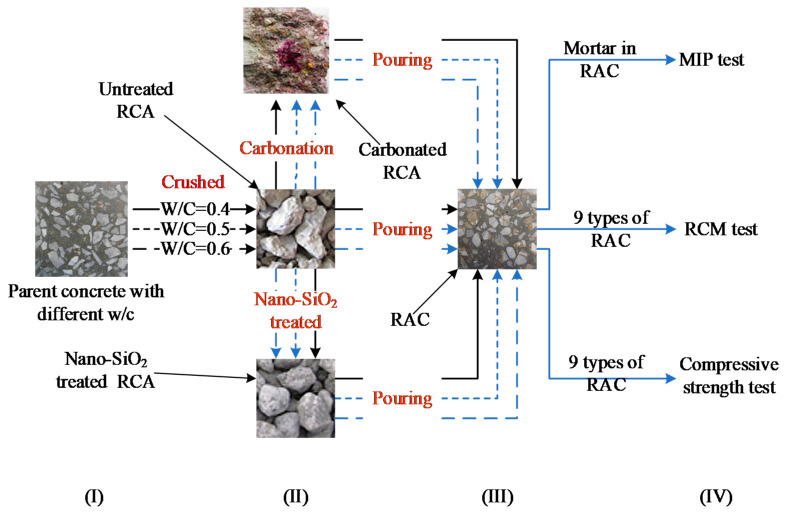
Flow diagram of the experiment.

As illustrated in Figure 1, the experimental program used in this study involves four steps: (I) Properties of parent concrete with different water-to-cement ratios (*W/C*_parent_) were characterized; (II) Parent concrete was crushed into RCAs, some of which was carbonated and nano-SiO_2_ slurry treated as new types of RCA, respectively; (III) Different types of RAC were prepared with nine types of RCA; (IV) The pore structure, diffusion coefficient, and compressive strength of RAC were measured experimentally.

### 2.1. Materials and Specimen Preparation 

#### 2.1.1. Materials

**Figure 2:**
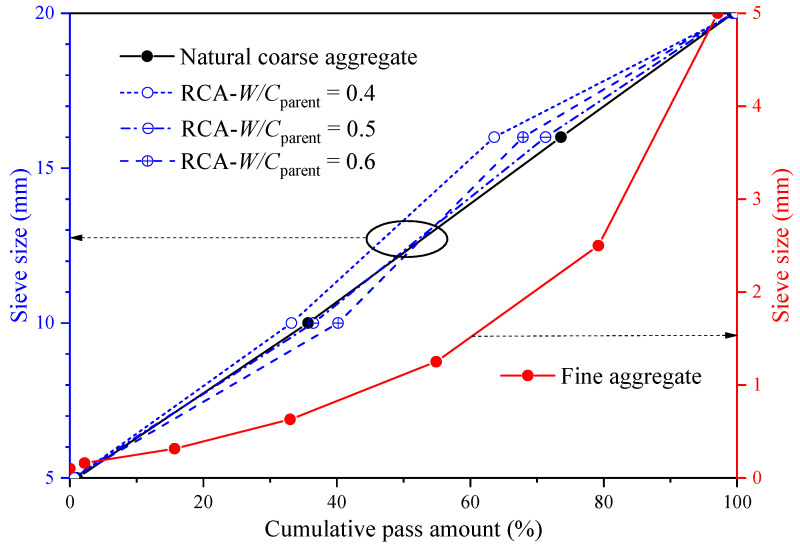
Grading curve of RCA.

The recycled concrete is made of nine types of RCA with different properties. The unmodified RCAs are derived from the crushed parent concrete, which is derived from the abandoned natural aggregate concrete tested by the standard pressure testing machine in the laboratory. The age of parent concrete varies from 56 to 128 days. The water-to-cement ratios of the three kinds of parent concrete are 0.4, 0.5, and 0.6, respectively, and their corresponding compressive strengths are 45, 31, and 24 MPa, respectively. Silica fume and nano-silica have similar chemical compositions, and their combination is beneficial to the mechanical properties and durability of concrete [21,22]. Therefore, an appropriate amount of silica fume (SiO_2_ 99.9%, particle size 2–10 μm) was used in the mix design. The mixture of parent concrete and recycled concrete is shown in Table 1; all of them used the same fine aggregate and cement. The volume fractions of RCA were the same for all types of RAC. The difference between them is mainly due to the different types of RCA and the amount of silica fume. The aggregate grading curves are shown in Figure 2. The cement used in this study was Portland cement (P.O 42.5), and its main components were C_3_S 55.7%, C_2_S 22.1%, C_3_A 5.1%, C_4_AF 16.8 and f-CaO 0.3%. The fine aggregate used was natural river sand, with water absorption of 0.83%, fineness modulus of 3.08 and apparent density of 2650 kg/m^3^. For parent concrete, the original natural aggregates used were limestone, with a diameter of 5–20 mm, apparent density of 2694 kg/m^3^ and water absorption of 0.85%, crushing index of 5.7%, and needle-like content of 6.7%. Both the coarse aggregates and fine aggregates used were in a saturated surface dry state. The concrete surface in the actual project is carbonated to varying degrees, but the carbonation depth of the concrete is only about 10 mm at the age of 50 years [23,24]. This means that most of the RCAs obtained by crushing the concrete in the actual project are not carbonated, similar to the RCAs obtained from the parent concrete.

#### 2.1.2. RCA Strengthening

**Figure 3:**
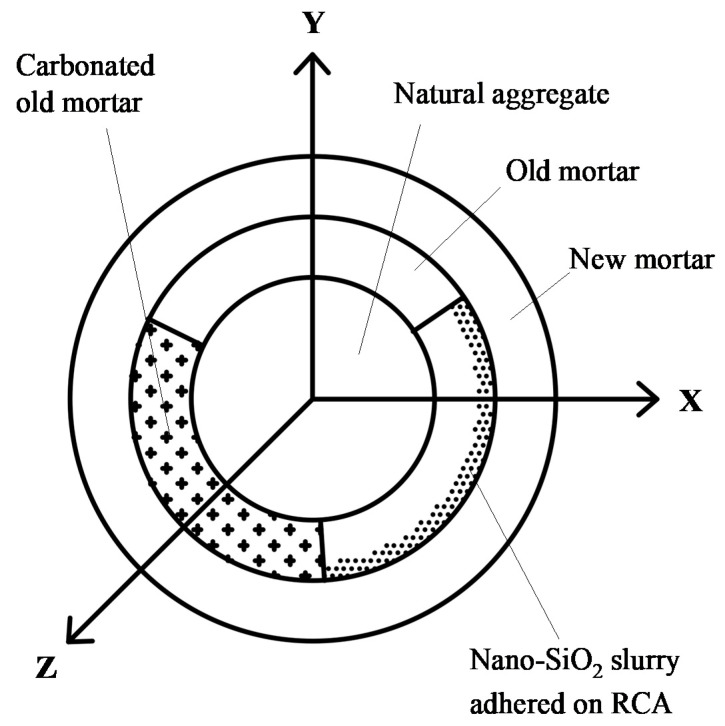
RAC model and strengthening schematic diagram of RCA.

**Figure 4:**
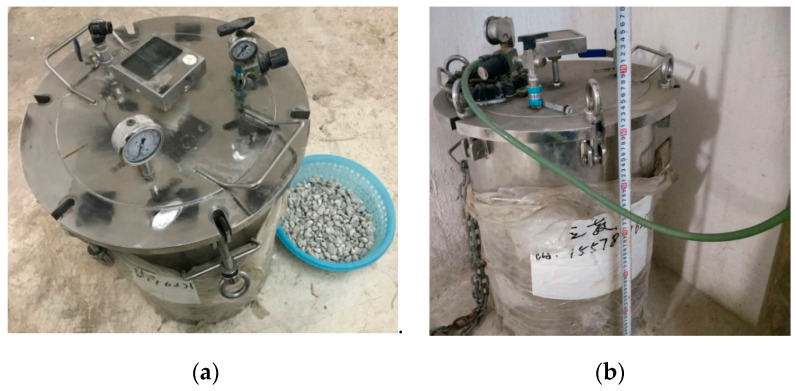
Experimental setup for CO_2_ curing: (**a**) the upper part of the carbonized cylinder and (**b**) the side of the carbonated cylinder.

For the RCAs with three water-to-cement ratios obtained by crushing the parent concrete with three water-to-cement ratios, a third of the three types of untreated RCAs were carbonated for 7 d, according to Ref. [14,15], and another third of them were nano-SiO_2_ slurry-treated according to Ref. [16,17,18]. The RAC model and strengthening schematic diagram of RCA after carbonation and nano-SiO_2_ slurry treatment is shown in Figure 3, and the particle size of nano silica was 15 ± 5 nm. As can be seen from the figure, the carbonation process strengthens almost all of the old mortar, which is consistent with the experimental results of Poon [25]. For the silica solution wrapping method, only the surface of the old mortar can be strengthened, and it is difficult for the slurry to enter the inside of the old mortar, which is consistent with the experimental results of Zhang [16]. For RCAs that are not reinforced in the above two ways, the old mortar did not change significantly, as shown graphically in Figure 3. For the carbonation of RCAs, the RCAs were washed in water and were then spread evenly on the outdoor ground for air drying in sunny weather until the RCA moisture content was between 30% and 50%. According to Ref. [25], an air-tight stainless-steel cylindrical chamber with a volume of 75 L was used for the carbonation of RCA, as shown in Figure 4. To remove the excess evaporated water from the specimens during the carbonation process [26], an appropriate amount of silica gel desiccant placed at the bottom of the chamber; then, the RCAs were put into the chamber. The container was vacuumed to −50 kPa by a vacuum pump, then high-pressure carbon dioxide (CO_2_) gas with a purity of over 99% was injected into the container, which is similar to that in Ref. [26]. The pressure of CO_2_ in the container was controlled at 15 kPa during carbonation. The temperature of the room where the container was located fluctuated within a range of 19–24 °C (M8).

The mix proportion of nano-SiO_2_ strengthening slurry was cement 1183 kg, water 592 kg, nano-SiO_2_ 24 kg, water reducer 18 kg and nano-dispersant 0.24 kg per m^3^. To obtain a suspension, nano-SiO_2_ and nano-dispersant were firstly added in water and then dispersed for 30 min by using the high-shear homogenizer (12,000 r/min, 300 W) and the ultrasonic disperser (40 kHz, 600 W). Next, nano-SiO_2_ suspension, cement and water-reducing agent were mixed for 6 min to obtain nano-SiO_2_ strengthening slurry. The air-dried RCAs were added to the nano-SiO_2_ strengthening slurry, soaked for 1 h, and stirred once every 5 min. Afterwards, the redundant nano-silica cement pastes were removed by using a square-hole sieve with a 4 mm edge length. Finally, the freshly prepared nano-SiO_2_ slurry-treated RCAs were evenly spread on a plastic film for 28 days with watering curing. 

#### 2.1.3. The Determination of Old Mortar Content

**Figure 5:**
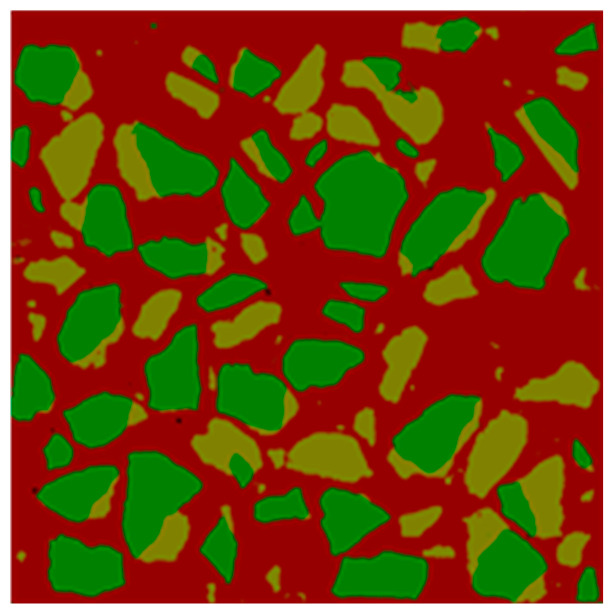
Image method for measuring the content of old mortar.

In this study, the apparent density method [27] and the image method were mainly used to test the apparent density of aggregates. The process of measuring the content of old mortar using the image method was as follows: First, the recycled concrete was polished by using a water-milling machine (5000 r/min, 900 W) and then scanned using a scanner (Canon CanoScan LiDE 700F, 4800 × 4800 dpi, Ho Chi Minh City, Vietnam) layer by layer to obtain the distribution map of RCAs at different depths. Then the deep learning method was used to identify and process the scanned graphics in order to obtain a clearer distribution map of RCA and mortar [28]. The manufacturing process is shown in Figure 5. In this figure, new mortar, old mortar and original natural aggregate in RCA are shown in red, yellow and green, respectively. For the RCAs obtained from the broken parent concrete, the old mortar content in the RCA estimated using the image method was 36%, and the error between it and the old mortar content obtained by the apparent density method was within 5%. For simplicity, the apparent density method was used to calculate the old mortar content of different types of RCAs.

#### 2.1.4. Properties of RCA

The properties of the RCAs were tested according to the Standard for technical requirements and test method of sand and crushed stone (or gravel) for ordinary concrete (JGJ/T 240-2011), as shown in Table 2. After being washed with water, the coarse aggregate had a mud content of 0.2–0.4% and a needle-like content of 7.8–8.2%.

#### 2.1.5. Specimen Preparation

To prepare the RAC, sand and cement were mixed at a low speed for 2 min in a compulsion-type concrete mixer (60 L, 1.5 kW), and then water, coarse aggregate and water reducer were slowly poured in and stirred for another 3 min to achieve excellent workability. Finally, the fresh concrete was poured into PVC plastic pipe with Ø100 × 300 mm to be used for chloride diffusion coefficient testing and into plastic molds to cast cubes of size 150 × 150 × 150 mm^3^ for compressive strength testing. A shaking table was used to reduce air bubbles in concrete and facilitate its compactness.

The concrete cubic specimens were de-molded after 24 h, and the concrete cylinder specimens were filled with about 50 mm depth of water for curing. All samples were cured in a standard curing room for the prescribed period. For any type of test, three identical samples were fabricated and tested. The coarse aggregates used in the mixture include untreated RCAs, carbonated RCAs and nano-SiO_2_ slurry-treated RCAs.

### 2.2. Test Methods

#### 2.2.1. Pore Structure

The concrete pore structures were measured using a mercury intrusion porosimeter (MIP, AutoPore IV 9500 American Michael Instruments Corp., Atlanta, GA, USA). The limit pressure of the mercury porosimeter is 33,000 psia, and the pore size of the sample can be tested ranges from 6 nm to 360 μm. To prepare the samples for the mercury intrusion porosimeter (MIP) test, a cutting machine was used to cut cylindrical concrete after the specified curing ages into slices with about 5 mm thickness, and the cutting part was located in the middle of the cylinder. New mortar and old mortar fragments with a size of about 5 mm were separately selected from the thin slices. To remove free water and facilitate vacuum drying, the debris of new mortar and old mortar was immersed in anhydrous ethanol, respectively. The pore structure of vacuum-dried samples was measured by using MIP.

#### 2.2.2. Chloride Diffusion in RAC

**Figure 6:**
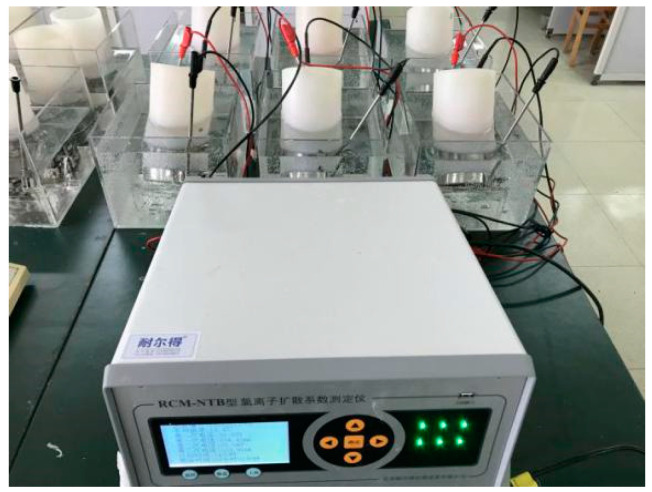
RCM test setup.

The details of the rapid chloride migration (RCM) test process corresponding to Figure 1 (IV) are described as follows: The RCM method proposed by Tang et al. [29] was applied in this work. The principle of this method is to generate chloride penetration through the sample by a solution concentration gradient and accelerate the movement of chloride using an electrical field. Since the thickness of concrete cover is generally less than 50 mm, it is reasonable to select 50-mm-thick samples for the chloride ion diffusion test. These sample disks with Ø100 × 50 mm were obtained from cylindrical samples using a cutting machine after curing for 28 days, and the cutting position was in the middle of the cylinder. The chloride migration test was performed on the sample disks, as shown in Figure 6.

## 3. Experimental Results and Discussions

### 3.1. Properties of Strengthened RCA 

In the process of coarse aggregate screening, due to the high-frequency vibration and shaking of the electric screen, the high-speed friction phenomenon occurred between the aggregate particles. In the process of crushing concrete, the particle size and strength of RCA exhibit great randomness. The above factors cause a slight difference in the gradation of different types of RCA, as shown in Figure 2. Because the concrete mix proportion is designed according to the volume method, that is to keep the same content of coarse aggregate in different types of concrete, the influence of aggregate gradation difference on concrete performance can be reduced as much as possible. It can be seen from Table 2 that, compared with unmodified RCA, in carbonation slurry, the water absorption of the carbonated RCAs with *W/C*_parent_ = 0.4, 0.5 and 0.6 decreased by 12%, 14% and 18%, respectively, and that of nano-SiO_2_ strengthened RCA with W/Cparent = 0.4, 0.5 and 0.6 decreased by 10%, 9% and 6%, respectively. Both the apparent density of RCA and that of the old mortar generally increased. The crushing index and the volume content of the old mortar of RCA did not change much. The reason for these phenomena may be that (1) the product of reaction of CO_2_ with hydration products (or unhydrated cement particles) adhered to the surface of untreated RCA can generate silica gels and solid CaCO_3_, which can fill the capillary pores in hardened cement paste [15], (2) the purpose of the nano-SiO_2_ slurry treatment is to coat a thin layer on the surface of RCA [30], (3) the porosity of RCA obtained from the parent concrete generally increases with increasing *W/C*_parent_ ratio, and (4) the remaining unhydrated cement particles in the old mortar may continue to hydrate.

### 3.2. The Pore Structure of Mortar in RAC

**Figure 7:**
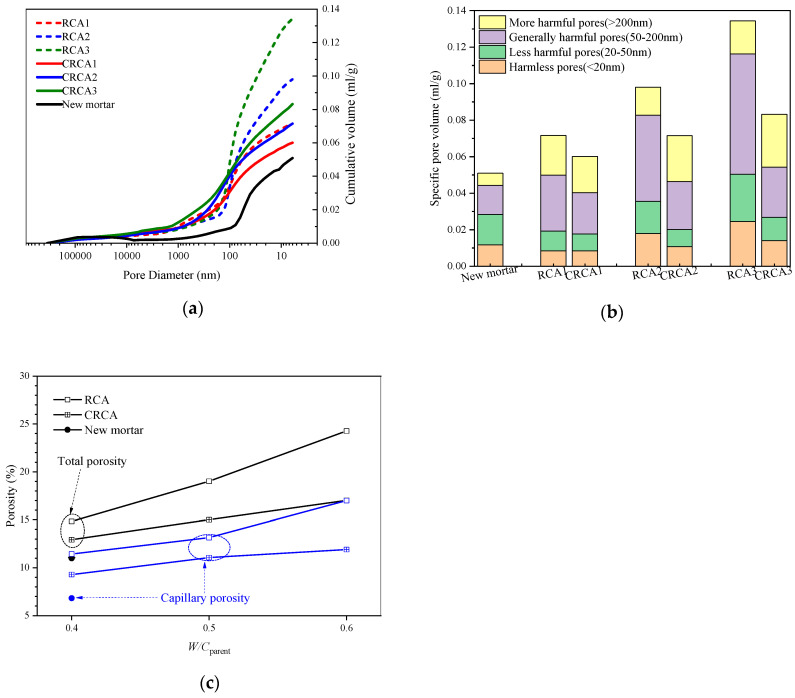
Pore structure of mortar in concrete: (**a**) cumulative curves of the pore size distribution, (**b**) pore volume distribution, and (**c**) porosity and capillary porosity of mortar.

The test results of MIP, including pore structure parameters such as pore size distribution and specific pore volume, are shown in Figure 7. Here, mortar in the untreated/carbonated RCA denotes the mortar adhered on the surface of untreated RCA and carbonated RCA, respectively. Because it is difficult to sample the old mortar of RCA after slurry wrapping, an MIP test was not carried out for it. The highest point of the cumulative curves represents the total specific pore volume of mortar in concrete.

It can be seen from Figure 7a that the cumulative specific pore volume increases with decreasing pore size, and the cumulative specific pore volume of new mortar is generally lower than that of old mortar. Porosity with pore sizes lower than 100 nm generally accounts for more than 50% of the total porosity, which may have a great impact on the chloride permeability of concrete. For the same *W/C*_parent_, when the pore size is less than 100 nm, the cumulative specific pore volume of mortar in carbonated RCAs is generally lower than that of mortar in untreated RCAs, and the difference between them gradually increases with decreasing pore size. The total specific pore volume of mortar in both the untreated RCAs and the carbonated RCAs increases with increasing *W/C*_parent_. The reason for this may be that the mortar in concrete with a higher *W/C*_parent_ ratio has a coarser pore structure. Furthermore, because the hydration products of ordinary cement, such as Ca(OH)_2_, C-S-H and ettringite were converted into calcium carbonate crystals [31], the porosities of mortar in the untreated RCAs are generally larger than that of mortar in carbonated RCAs for the same *W/C*_parent_. Additionally, the porosity of old mortar in the carbonated RCAs is still larger than that of the new mortar in RAC due to the crushing effect or damages [32] when the RCA is obtained from the parent concrete. 

According to the effect of different pore size on chloride diffusion in concrete [4], pores in concrete can be divided into more harmful pores (>200 nm), general harmful pores (50–200 nm), less harmful pores (20–50 nm) and harmless pores (<20 nm). According to this classification, the pore system of mortar in concrete was divided into four ranges, as shown in Figure 7b. It can be seen from the figure that the number of harmful pores of mortar in the untreated RCAs increases significantly with increasing *W/C*_parent_ ratio. However, all the numbers decreased to very low levels after being carbonated. For example, the harmful pore specific pore volume of mortar in carbonated RCAs with *W/C*_parent_ ratio = 0.4, 0.5 and 0.6 was reduced by 19%, 18% and 33%, respectively, compared with untreated RCAs.

Porosity generally refers to the volume ratio of open holes in the material to the volume of the whole material. Capillary pores are the primary channel for chloride ion diffusion, and pore sizes of 30–10,000 nanometers [33] are generally defined as capillary pores. The porosity is calculated by multiplying the total specific pore volume by Bulk Density at 0.52 psia, and so on. The porosity and capillary porosity of mortar in the RCA obtained from Figure 7a is shown in Figure 7c. It indicates that both the porosity and capillary porosity of mortar in the untreated and carbonated RCAs increase with increasing *W/C*_parent_ ratio. Furthermore, the carbonation effect of mortar in the RCA is similar to that in Ref. [34], as shown in Figure 7c. Specifically, compared with the mortar in the untreated RCA, the porosity of the mortar in the carbonated RCA decreases by 13%, 21% and 30%, and the capillary porosity of the mortar in the carbonated RCA decreases by 19%, 16% and 30% for concrete with *W/C*_parent_ = 0.4, 0.5 and 0.6, respectively. These phenomena indicate that the effect of carbonation treatment in refining the pore structure of concrete increases with increasing *W/C*_parent_.

### 3.3. Compressive Strength of RAC

**Figure 8:**
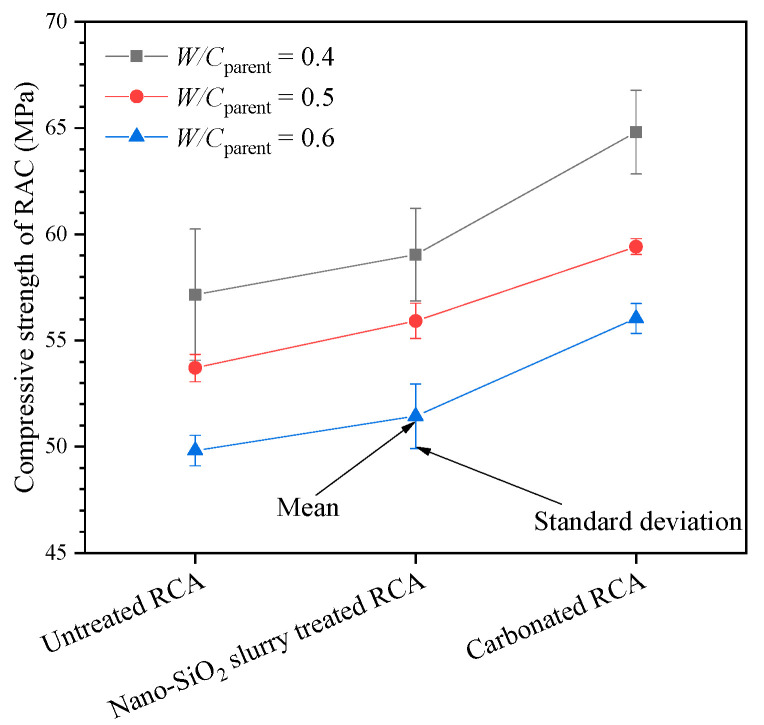
The compressive strength of RAC containing different types of RCAs.

The 28 d compressive strengths of RAC containing different types of RCA are summarized in Figure 8. The legend “*W/C*_parent_ = 0.4” denotes the compressive strength of the RAC containing RCA resulted from the parent concrete with *W/C* = 0.4, as shown in Figure 1, and so on. It can be seen from the figure that the compressive strength of RAC varies from 50 to 65 MPa. The water-to-cement ratio of recycled concrete is 0.4, and the Portland cement is mixed with 10% primary micro silica powder, which can largely fill the micropore of concrete and improve the strength of concrete. For the same type of aggregate, the concrete compressive strength increases with the decrease of *W/C*_parent_. Similar results can be found in Ref. [15]. In particular, the compressive strength of the concrete containing the untreated RCA with *W/C*_parent_ = 0.4 and 0.5 is 14.7% and 7.8% larger than that with *W/C*_parent_ = 0.6, respectively. The compressive strength of the concrete containing the nano-SiO_2_ slurry-treated RCA with *W/C*_parent_ = 0.4 and 0.5 is 14.8% and 8.7% higher than that with *W/C*_parent_ = 0.6, respectively. The compressive strength of the concrete containing the carbonated RCA with *W/C*_parent_ = 0.4 and 0.5 is 15.7% and 6% higher than that with *W/C*_parent_ = 0.6, respectively. The reason for this is that the properties of RCA resulting from the concrete with *W/C*_parent_ of 0.6 are worse than others, as shown in Table 2. For example, regardless of the treatment for the RCAs, the crushing value and porosity of RCAs resulting from concrete with *W/C*_parent_ = 0.6 are more significant than those with *W/C*_parent_ = 0.4 or *W/C*_parent_ = 0.5. The reason for this may be that the compressive strength of concrete increases with decreasing porosity [35].

Compared with the RAC containing the untreated RCAs, the compressive strength of the RAC containing the nano-SiO_2_ slurry-treated RCA increases by 3.3%, 4.1% and 3.2%, and that containing the carbonated RCA increases by 13%, 11% and 13% for the concrete with *W/C*_parent_ = 0.4, 0.5 and 0.6, respectively. This phenomenon is similar to the experimental results in Refs. [30,36,37]. The nano-SiO_2_ slurry treatment coated a thin silica layer on the surface of RCA, which may enhance the ITZ between the new mortar and the RCA via pozzolanic reaction [30,38]. The carbonation treatment filled the larger pores, which resulted in the whole microstructure becoming denser [31].

### 3.4. Chloride Diffusion of RAC

**Figure 9:**
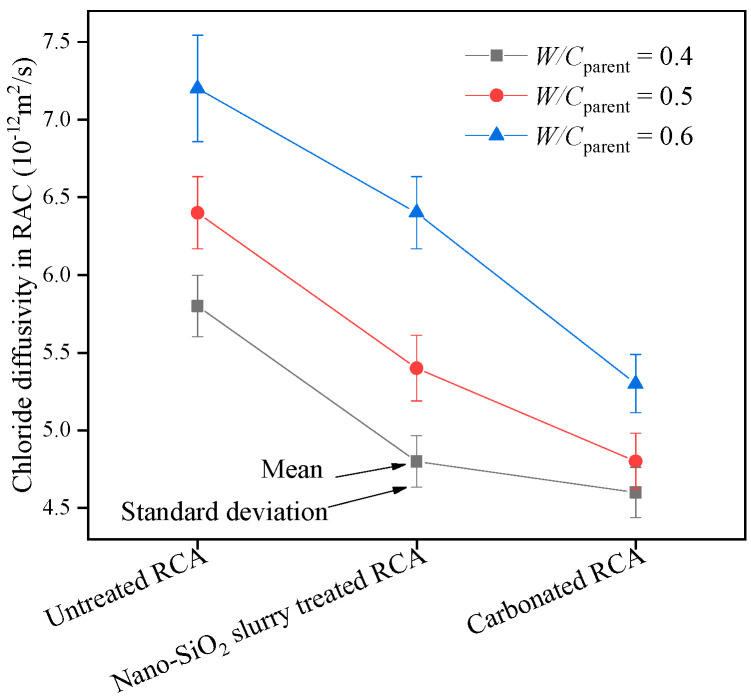
The value of *D*_RAC_ with different types of RCAs.

The chloride ion diffusion coefficient in RAC (*D*_RAC_) containing different types of RCAs is summarized in Figure 9. As depicted in Figure 9, *D*_RAC_ varies from 4.6 to 7.2 × 10^−12^ m^2^/s. *D*_RAC_ of RAC containing the untreated RCA with *W/C*_parent_ = 0.5 and 0.6 is 10% and 24% higher than that with *W/C*_parent_ = 0.4, respectively; *D*_RAC_ of RAC containing the nano-SiO_2_ slurry-treated RCA with *W/C*_parent_ = 0.5 and 0.6 is 13% and 33% larger than that with *W/C*_parent_ = 0.4, respectively; *D*_RAC_ of RAC containing the carbonated RCA with *W/C*_parent_ = 0.5 and 0.6 is 4% and 15% higher than that with *W/C*_parent_ = 0.4, respectively.

The reason for these increases is that the porosity and capillary porosity of mortar in the RCA resulting from concrete with *W/C*_parent_ of 0.4 are lower than the others, as shown in Figure 7, and the chloride ion penetration of concrete increases with increasing capillary porosity [39,40,41].

Compared with RAC containing untreated RCA, the *D*_RAC_ of concrete containing the nano-SiO_2_ slurry-treated RCA decreases by 17%, 16% and 11%, and that of RAC containing the carbonated RCA decreases by 21%, 25% and 26% for *W/C*_parent_ = 0.4, 0.5 and 0.6, respectively. This indicates that the carbonation of RCAs can decrease the chloride diffusion coefficient of concrete, especially for the RCAs resulted from the concrete with larger *W/C*_parent_. This phenomenon is similar to the experimental results in Ref. [31], and the reason for this may be that the surface treatment of the RCA with nano-SiO_2_ slurry enhanced the new interfacial transition zone (ITZ) in concrete, which contributes to the prevention of chloride ion migration [18,30], and the carbonation-treated RCAs have lower water absorption than the untreated RCA [25], as shown in Table 2. In addition, it is further discussed that for the natural coarse aggregate concrete, in the concrete, the aggregate itself hinders the chloride ion transmission, but the interface transition zones between aggregate and cement can accelerate the chloride ion transmission. When the total volume content of aggregate is constant, increasing the particle size of aggregate will result in a variety of results: (1) the decrease of aggregate total specific surface area leads to the decrease of total interfacial transition zone, which reduces the chloride ion transmission path, and (2) the degree of single aggregate necessary to block chloride ion diffusion becomes stronger. It is certain that increasing aggregate size will increase the dispersion of chloride ion diffusion coefficient test results under the condition of a constant total aggregate content due to the random distribution of aggregate in concrete. Therefore, the aggregate size selected in this study is no more than 20 mm, as shown in Figure 2. For RAC, the old mortar in the RCA will accelerate chloride ion transmission, while the original natural aggregate in the RCA hinders chloride ion transmission, which is more complex than the natural aggregate concrete, so the influence of the content of the old mortar in RCA on the chloride ion diffusion needs to be further considered.

## 4. Prediction of the Chloride Diffusion Coefficient in Recycled Concrete

### 4.1. Three-Phase Model for the Prediction of D_RAC_

To predict the chloride ion diffusion coefficient, concrete can be idealized using various models [10,20,42]. The homogenization model [42,43] is often used to predict the chloride diffusion coefficient in hardened cement paste or concrete. If concrete is regarded as a multi-phase composite material, the property and content of ITZ are one of the important factors affecting the chloride diffusion in concrete. Since the size of the coarse aggregate is much larger than that of fine aggregate, the content of ITZ caused by RCA can be negligible [44]. Therefore, RAC can be regarded as a multi-phase composite material consisting of new mortar, old mortar attached to RCA surface and original natural aggregate. Assuming that the chloride diffusivity of natural aggregate is 0, a three-phase chloride diffusion coefficient model of RAC derived from Refs. [44] can be given as follows,

(1) $$D_{\mathrm{RAC}}=a\cdot D_{\mathrm{nm}}\left[1-\frac{3\left(3V_{\mathrm{ra}}(1-R_{\mathrm{om}})+2(1-q)R_{\mathrm{om}}V_{\mathrm{ra}}\right)}{4+2\cdot q+2(1-R_{\mathrm{om}})(1-q)+3V_{\mathrm{ra}}(1-R_{\mathrm{om}})+2(1-q)R_{\mathrm{om}}V_{\mathrm{ra}}}\right]$$

where *R*_om_ and *D*_om_ are the adhesive rate and chloride diffusivity of old mortar; *D*_nm_ is the chloride diffusivity of new mortar; *q* = *D*_om_/*D*_nm_; *V*_ra_ is the RCA volume fractions.

Considering uncertainties such as aggregate gradation and shape, the coefficient *a* is a parameter to be calibrated. According to Refs. [45,46], the relationships between chloride diffusivity and porosity of mortar are

(2) $$D_{\mathrm{nm}}=D_{0}\cdot \left[0.001+0.07\cdot \phi_{\mathrm{capnm}}^{2}+1.8\cdot Heaviside\left(\phi_{\mathrm{capnm}}^{}-\phi_{\mathrm{crinm}}^{}\right)\cdot {\left(\phi_{\mathrm{capnm}}^{}-\phi_{\mathrm{crinm}}^{}\right)}^{2}\right]$$

(3) $$D_{\mathrm{om}}=D_{0}\cdot \left[0.001+0.07\cdot \phi_{\mathrm{capom}}^{2}+1.8\cdot Heaviside\left(\phi_{\mathrm{capom}}^{}-\phi_{\mathrm{criom}}^{}\right)\cdot {\left(\phi_{\mathrm{capom}}^{}-\phi_{\mathrm{criom}}^{}\right)}^{2}\right]$$

where *D*_0_ is the chloride diffusivity in bulk water at 25 °C; $\phi_{\mathrm{capnm}}^{}$ and $\phi_{\mathrm{capom}}$ are the capillary porosity of new mortar and old mortar, respectively; and *ϕ*_crinm_ and *ϕ*_criom_ are the critical porosity of new mortar and old mortar, respectively. A porosity of 0.18 is defined as the critical porosity [47], and the capillary pore space is disconnected at critical porosity.

Compared with testing the pore structure of aggregate, it is easier to measure the water absorption of RCA in practical engineering. Therefore, it is necessary to establish the relationship between the water absorption of RCA and the chloride diffusion coefficient of RAC. When the RCA is regarded as a two-phase composite composed of old mortar and original natural aggregate, the following expression can be obtained:

(4) $$R_{\mathrm{sopRCA}}=\frac{M_{\mathrm{waterInOM}}+M_{\mathrm{waterInOA}}}{M_{\mathrm{RCA}}}$$

(5) $$R_{\mathrm{sopOM}}=\frac{M_{\mathrm{waterInOM}}}{M_{\mathrm{dryOM}}}$$

(6) $$R_{\mathrm{sopOA}}=\frac{M_{\mathrm{waterInOA}}}{M_{\mathrm{dryOA}}}$$

(7) $$M_{\mathrm{dryOA}}=M_{\mathrm{RCA}}\left(1-R_{\mathrm{OM}}\right)$$

(8) $$M_{\mathrm{dryOM}}=M_{\mathrm{RCA}}R_{\mathrm{OM}}.$$

where, *R*_sopRCA_, *R*_sopOM_ and *R*_sopOA_ are the water absorption of RCA, old mortar and original natural aggregate, respectively. *M*_waterOM_ and *M*_waterInOA_ are the water content of old mortar and original natural aggregate in the saturated surface dry state, respectively. *M*_RCA_, *M*_dryOM_ and *M*_dryOA_ are the mass of RCA, old mortar and original natural aggregate in the dry state, respectively. *R*_OM_ is the content of old mortar obtained by mass ratio, that is, the mass of old mortar divided by the mass of RCA.

Based on Equations (4)–(8), the following formula can be obtained:

(9) $$R_{\mathrm{sopOM}}=-\frac{-R_{\mathrm{sopRCA}}+R_{\mathrm{sopOA}}}{R_{\mathrm{OM}}}+R_{\mathrm{sopOA}}$$

Because *R*_OM_ is calculated by mass ratio, it should be converted to *R*_om_ calculated by volume ratio, and the relationship between them is

(10) $$R_{\mathrm{OM}}=\frac{\rho_{\mathrm{OM}}}{\rho_{\mathrm{RCA}}}R_{\mathrm{om}}$$

where $\rho_{\mathrm{OM}}$ and $\rho_{\mathrm{RCA}}$ are the apparent density of old mortar and RCA, respectively.

**Figure 10:**
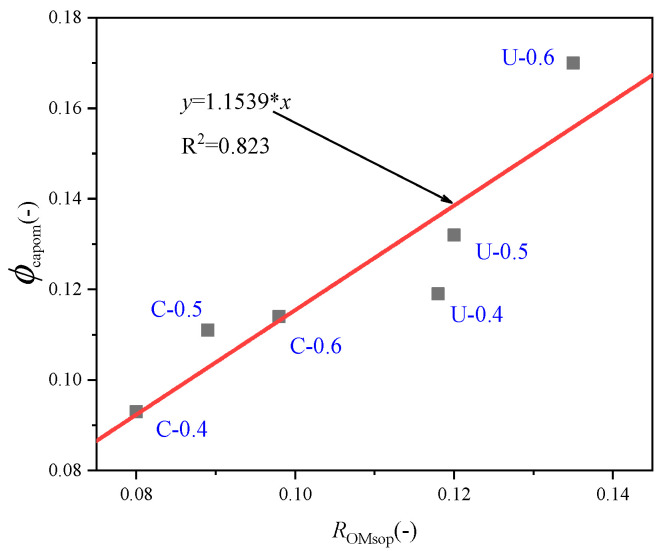
The relationship between water absorption and capillary porosity of old mortar.

To obtain the relationship between the capillary porosity and water absorption of old mortar, a part of the broken old mortar is carbonated, and its capillary porosity is obtained by the mercury intrusion method, and its water absorption is obtained by the immersion method. The relationship between the water absorption and capillary porosity of the old mortar is shown in Figure 10. 

Since the old mortar coated with nano cement is difficult to remove, and the removal process may affect the original pore structure, its pore structure and water absorption are not tested. In the figure, for example, U-0.4 refers to old mortar without carbonation with a water-to-cement ratio of 0.4, while C-0.4 refers to old mortar with carbonation with a water-to-cement ratio of 0.4, and so on. The solid red line denotes the linear regression of these data. It can be seen from the figure that there is a good linear correlation between the capillary porosity of the old mortar and its water absorption, and the relationship between them is

(11) $$\phi_{\mathrm{capom}}=1.15R_{\mathrm{sopOM}}$$

By substituting Equation (10) into Equation (9), Equation (9) into Equation (11), Equation (11) into Equation (3), and Equation (2) and Equation (3) into Equation (1), we can obtain the function relationship as follows:

(12) $$D_{\mathrm{RAC}}=a\cdot f\left(D_{0},R_{\mathrm{sopRCA}},R_{\mathrm{sopOA}},V_{\mathrm{ra}},\frac{\rho_{\mathrm{OM}}}{\rho_{\mathrm{RCA}}},R_{\mathrm{om}},\phi_{\mathrm{criom}}^{},\phi_{\mathrm{crinm}}^{},\phi_{\mathrm{capnm}}^{}\right)$$

Equation (12) shows the functional relationship between the chloride diffusion coefficient of RAC and some parameters such as water absorption of RCA.

### 4.2. Test Verification

**Figure 11:**
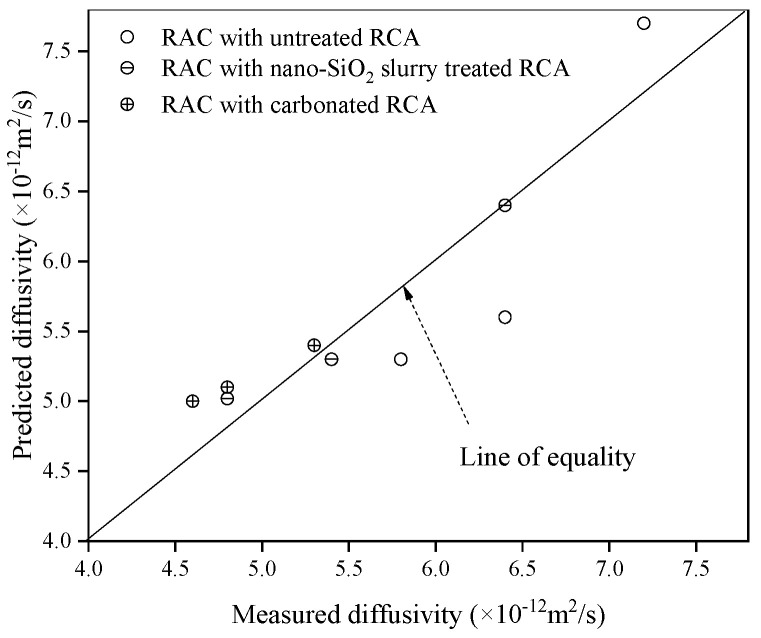
Comparison between the test results and theoretical prediction of RAC.

To determine the validity of Equation (12), it is necessary to calculate the undetermined coefficient according to the existing experimental data. For all types of RCA, $\phi_{\mathrm{capnm}}^{}$= 6.8%, *V*_ra_ = 40% and $R_{\mathrm{sopOA}}$= 0.85%. Let $\phi_{\mathrm{criom}}$ = 0.18, *ϕ*_crinm_ = 0.18 [47] and *D*_0_ = 2.03 × 10^−9^ m^2^/s based on Ref. [46], $R_{\mathrm{sopRCA}}$, $\rho_{\mathrm{RCA}}$, $\rho_{\mathrm{OM}}$ and *R*_om_ are shown in Table 2. The chloride diffusion coefficient of RAC is shown in Figure 9. By substituting the above data into Equation (12), the specific value of *a* corresponding to different concrete can be calculated. They are generally between 2.2 and 2.7. If the value *a* = 2.38 is selected as the specific value of Equation (12), the theoretical value of the chloride diffusion coefficient of different types of RAC can be calculated. The comparison between the theoretical value and the experimental value is shown in Figure 11. The results show that the theoretical prediction of chloride diffusivity in RAC is in good agreement with the experimental results. Therefore, Equation (12) can be used to predict $D_{\mathrm{RAC}}$ and to perform parametric analysis.

### 4.3. Parameter Analysis 

**Figure 12:**
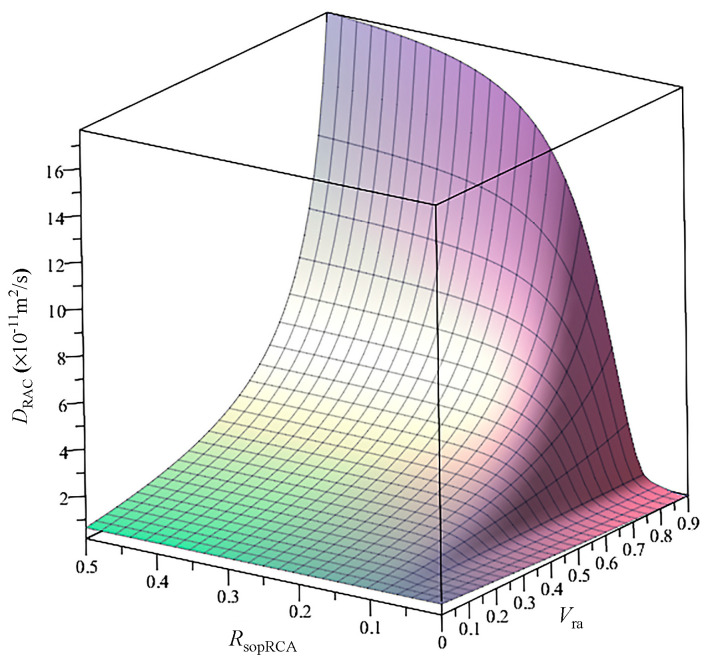
*D*_RAC_ (×10^−1^^1^ m^2^/s) varying with water absorption and volume fractions of RCA.

Since it is easy to determine $R_{\mathrm{sopRCA}}$, *R*_om_ and $V_{\mathrm{ra}}$ by experiments, their effect on the $D_{\mathrm{RAC}}$ is analyzed by assuming $R_{\mathrm{om}}$ = 0.38, $\phi_{\mathrm{capnm}}^{}$ = 0.07, *a* = 0.38, $R_{\mathrm{sopOA}}$ = 0.85%, $\rho_{\mathrm{RAC}}=2594$ kg/m^3^ and $\rho_{\mathrm{OM}}=2193$ kg/m^3^ . Thus, $D_{\mathrm{RAC}}$ is calculated using Equation (12), as shown in Figure 12. It can be seen from the figure that $D_{\mathrm{RAC}}$ varies with $R_{\mathrm{sopRCA}}$ and $V_{ra}$. For a given $V_{ra}$, $D_{\mathrm{RAC}}$ increases with increasing $R_{\mathrm{sopRCA}}$ and the speed of increase grows with increasing $V_{ra}$. When $R_{\mathrm{sopRCA}}$ varies in the range from 0% and 6.2%, $D_{\mathrm{RAC}}$ decreases gradually with increasing $V_{ra}$. When $R_{\mathrm{sopOA}}$ is larger than 6.2%, $D_{\mathrm{RAC}}$ increases with increasing $V_{ra}$, and the increased speed of $D_{\mathrm{RAC}}$ increases with increasing $R_{\mathrm{sopRCA}}$. For example, the numbers of *D*_RAC_ (× 10^−1^^1^ m^2^/s) corresponding to different $R_{\mathrm{sopRCA}}$ and $V_{ra}$ are shown in Table 3. This phenomenon can be explained as follows: in the process of crushing, due to the influence of internal damage, the porosity of mortar will increase [32], which can lead to increasing water absorption, while the water absorption of the original natural aggregate in the RCA is almost zero. Increasing the content of RCA can simultaneously increase the two phases of RCA and decrease the content of new mortar. When $R_{\mathrm{sopRCA}}$ is small enough, chloride ions cannot easily diffuse in the RCA; increasing *V*_ra_ can decrease the content of new mortar and the chloride diffusivity of RAC. When $R_{\mathrm{sopRCA}}$ is large enough, chloride ions can easily diffuse in the RCA, while increasing *V*_ra_ can increase the content of RCA and the chloride diffusivity of RAC.

**Figure 13:**
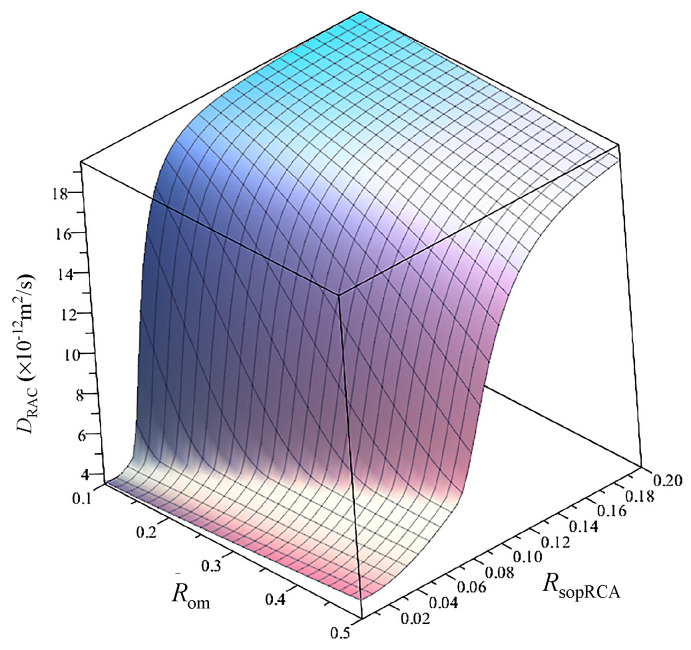
*D*_RAC_ (×10^−12^ m^2^/s) changing with water absorption of RCA and old mortar content in RCA.

To analyze the effects of $R_{\mathrm{om}}$ and $R_{\mathrm{sopRCA}}$ on $D_{\mathrm{RAC}}$, $V_{\mathrm{ra}}$ = 0.4, calculated from Table 1, is used. The other parameters are the same as those in Section 4.2. Then the value of $D_{\mathrm{RAC}}$ can be determined by using Equation (12), as shown in Figure 13. The figure shows that $D_{\mathrm{RAC}}$ increases with increasing $R_{\mathrm{sopRCA}}$, and the increased speed is affected by $R_{\mathrm{om}}$. When $R_{\mathrm{om}}$ is small, $D_{\mathrm{RAC}}$ increases slowly and then rapidly with increasing $R_{\mathrm{sopRCA}}$. When $R_{\mathrm{om}}$ is large, *D*_RAC_ increases slowly over a long distance, and then becomes larger and more stable. For example, the numbers of *D*_RAC_ (× 10^−1^^2^ m^2^/s) corresponding to different $R_{\mathrm{om}}$ and $R_{\mathrm{sopRCA}}$ are shown in Table 4. This situation can be interpreted as increasing $R_{\mathrm{sopRCA}}$ directly leading to an increase in the capillary porosity of the old mortar, and then results in increasing diffusion coefficient of old mortar. When $R_{\mathrm{sopRCA}}$ increases by the same amount, the smaller the $R_{\mathrm{om}}$ is, the faster the diffusion coefficient of the old mortar will increase, and then the faster $D_{\mathrm{RAC}}$ will increase. As the content of RCA is constant, $D_{\mathrm{RAC}}$ tends to be stable with increasing $R_{\mathrm{sopRCA}}$.

## 5. Conclusions

The properties of the parent concrete have an important influence on those of recycled aggregate concrete (RAC). The recycled coarse aggregates (RCAs) were obtained by crushing the parent concrete with different water cement ratio (*W/C*_parent_), and then the RCAs were strengthened by the carbonation method and the nano cement slurry wrapping method respectively, and then different types of RCAs were obtained and subsequently poured into the RAC. This study mainly analyzes the influence of *W/C*_parent_ on the properties of RCA and RAC. The conclusions are drawn as follows:

The *W/C*_parent_ can affect the properties of RCAs. The water absorption of carbonated RCA with *W/C*_parent_ = 0.4, 0.5 and 0.6 decreases by 12%, 14% and 18%, respectively, and that of nano-SiO_2_-strengthened RCA with W/Cparent = 0.4, 0.5 and 0.6 decreases by 10%, 9% and 6%, respectively. Compared with the mortar in the untreated RCA, the porosity of the mortar in the carbonated RCA decreases by 13%, 21% and 30%, and the capillary porosity of the mortar in the carbonated RCA decreases by 19%, 16% and 30% for the concrete with *W/C*_parent_ = 0.4, 0.5 and 0.6, respectively.

Regardless of their treatment method, with increasing *W/C*_parent_, the RAC compressive strength decreases, while the chloride diffusion coefficient of RAC increases. Compared with the RAC containing the untreated RCAs, the compressive strength of the RAC containing the nano-SiO_2_ slurry-treated RCA increased by 3.3%, 4.1% and 3.2%, and that containing the carbonated RCA increases by 13%, 11% and 13% for the concrete with *W/C*_parent_ = 0.4, 0.5 and 0.6, respectively; the *D*_RAC_ of concrete containing the nano-SiO_2_ slurry-treated RCA decreased by 17%, 16% and 11%, and that of RAC containing the carbonated RCA decreased by 21%, 25% and 26% for *W/C*_parent_ = 0.4, 0.5 and 0.6, respectively.

The value of *D*_RAC_ is affected by many factors including the capillary porosities of new mortar, RCA water absorption (*R*_sopRCA_), the volume fractions of RCA (*V*_ra_) and old mortar content in RCA by volume ratio (*R*_om_) and so on. With increasing *V*_ra_, *D*_RAC_ decreases slowly when *R*_sopRCA_ is low enough, while it increases rapidly when *R*_sopRCA_ becomes large enough.

To ensure the quality of concrete construction, it is suggested to reduce the *R*_sopRCA_ and *R*_om_ or strengthen the RCA by using a carbonated method or nano-SiO_2_ slurry treatment of RCA. If CO_2_ produced in cement production process is used to strengthen RCAs, good environmental effect will be achieved.

## Figures and Tables

**Table 1:** Concrete mix proportion (kg/m^3^).

No.	Coarse Aggregate Type	Cement	Silica Fume	Water	Coarse Aggregate	Fine Aggregate	Water Reducing Agent
NAC1	NCA	465	0	186	1054	705	5
NAC2	NCA	408	0	204	1054	705	4
NAC3	NCA	364	0	218	1054	705	4
RAC1	RCA1	405	45	180	957	705	4
RAC2	RCA2	405	45	180	948	705	4
RAC3	RCA3	405	45	180	935	705	4
CRAC1	CRCA1	405	45	180	968	705	4
CRAC2	CRCA2	405	45	180	962	705	4
CRAC3	CRCA3	405	45	180	954	705	4
NRAC1	RCA1-ns	405	45	180	965	705	4
NRAC2	RCA2-ns	405	45	180	954	705	4
NRAC3	RCA3-ns	405	45	180	939	705	4

NCA denotes natural coarse aggregate; NAC1, NAC2 and NAC3 denotes NCA concrete with water-to-cement ratios 0.4, 0.5 and 0.6, respectively; RCA1 denotes RCA crushed from NAC1; CRCA1 denotes carbonated RCA1; RCA1-ns denotes nano-SiO_2_-strengthened RCA1, and so on.

**Table 2:** Properties of RCA.

No.	*R*_sopRCA_ (%)	*ρ*_OM_ (kg/m^3^)	*ρ*_RCA_ (kg/m^3^)	*R*_om_ (%)	*R*_crushing_ (%)
RCA1	5	2190	2594	37.2	17.1
RCA2	5.6	2181	2589	38.2	17.8
RCA3	6.5	2095	2583	38.3	18.4
CRCA1	4.4	2255	2603	37.8	16.3
CRCA2	4.8	2248	2601	38.8	16.2
CRCA3	5.3	2244	2597	38.5	17.1
RCA1-ns	4.5	2213	2598	37.7	16.1
RCA2-ns	5.1	2195	2591	39.0	17.2
RCA3-ns	6.1	2120	2581	38.8	17.3

*R*_om_, *R*_sopRCA_ and *R*_crushing_ represent the old mortar content obtained according to the apparent density method, the water absorption rate of the RCA, and the crushing index of the coarse aggregate, respectively; *ρ*_RCA_ and *ρ*_OM_ represent the apparent density of the RCA and the old mortar in the RCA, respectively.

**Table 3:** The *D_RAC_* (×10^−1^^1^ m^2^/s) corresponding to Figure 12.

	$V_{ra}\;=\;0\%$	$V_{ra}\;=\;30\%$	$V_{ra}\;=\;60\%$	$V_{ra}\;=\;90\%$
$R_{\mathrm{sopRCA}}$ = 1%	0.65	0.46	0.31	0.18
$R_{\mathrm{sopRCA}}$ = 7%	0.65	0.80	0.99	1.20
$R_{\mathrm{sopRCA}}$ = 17%	0.68	1.43	3.28	12.8

**Table 4:** The *D*_RAC_ (×10^−12^ m^2^/s) corresponding with Figure 13.

	$R_{\mathrm{RCAsop}}\;=\;0\%$	$R_{\mathrm{RCAsop}}\;=\;5\%$	$R_{\mathrm{RCAsop}}\;=\;10\%$	$R_{\mathrm{RCAsop}}\;=\;20\%$
$R_{\mathrm{om}}$ = 10%	3.6	16.2	19.1	19.5
$R_{\mathrm{om}}$ = 50%	4.4	5.4	12.4	18.8

## Notation and Abbreviations

RAC	recycled aggregate concrete
*D*_RAC_	the chloride diffusion coefficient in RAC
ITZ	interfacial transition zone
NCA	natural coarse aggregate
RCA	recycled coarse concrete
CRCA	carbonated RCA
$D_{\mathrm{rm}}$	the chloride diffusion coefficient in old mortar
$V_{\mathrm{ra}}$	the volume fraction of recycled coarse concrete
*ϕ*_capnm_	capillary porosity of the new mortar
*ϕ*_crinm_	critical porosity of the new mortar
*ϕ*_capom_	capillary porosity of the old mortar;
*ϕ*_criom_	critical porosity of the old mortar;
*R*_sopRCA_	water absorption of RCA
*R*_sopOM_	water absorption of old mortar
*R*_sopOA_	water absorption of original natural aggregate
*M*_waterOM_	the water content of old mortar in the saturated surface dry state
*M*_waterInOA_	the water content of original natural aggregate in the saturated surface dry state
*M*_dryOM_	mass of old mortar in the dry state
*M*_dryOA_	mass of original natural aggregate in the dry state
*M*_RCA_	mass of RCA in the dry state
*M*_dryOM_	mass of old mortar in the dry state
*M*_dryOA_	mass of original natural aggregate in the dry state
*R*_OM_	old mortar content in RCA by mass ratio
$R_{\mathrm{om}}$	old mortar content in RCA by volume ratio
$\rho_{\mathrm{OM}}$	the apparent density of an old mortar
$\rho_{\mathrm{RCA}}$	the apparent density of RCA
*W/C*_parent_	the water-to-cement ratio of parent concrete
